# Genetic Dissection of Major Rice QTLs for Strong Culms and Fine Mapping of *qWS5* for Breeding Application in Transplanted System

**DOI:** 10.1186/s12284-024-00723-x

**Published:** 2024-07-12

**Authors:** Zhong Bian, Dongping Cao, Yiting Zou, Dong Xie, Wenshu Zhuang, Zixing Sun, Nana Mou, Yangyang Sun, Changquan Zhang, Qianfeng Li, Qiaoquan Liu, Lin Zhang

**Affiliations:** 1 Joint International Research Laboratory of Agriculture and Agri-Product Safety of the Ministry of Education/Jiangsu Key Laboratory of Crop Genomics and Molecular Breeding, Yangzhou University, Yangzhou, China; 2 Jiangsu Co-Innovation Center for Modern Production Technology of Grain Crops/Jiangsu Key Laboratory of Crop Genetics and Physiology, Yangzhou University, Yangzhou, China

**Keywords:** Rice, Lodging resistance, Stem diameter, QTL, *qWS5*, Breeding

## Abstract

**Background:**

Rice is one of the major staples that feeds about one half of the global populations, and it is important to identify the genetic loci for the traits related to yield improvement. Lodging will cause severe yield loss when it happens, and stem diameter has been characterized as an important trait for lodging resistance. However, most QTLs for stem diameter have not been finely dissected due to their sensitivity to environmental fluctuation.

**Result:**

In this study, we performed QTL analysis for stem diameter using populations derived from Nipponbare (NIP) and strong culm variety YYP1, and confirmed the single and combined effect of three major QTLs by recombinant inbred lines (RILs). Based on the QTL location, we found that *qWS5* is a novel QTL not well characterized before. To finely dissect the novel locus, several recombinant heterogeneous inbred families (HIFs) were selected from the RILs for linkage analysis and their derived nearly isogenic lines (NILs) were subjected to detailed trait investigation throughout different years. The HIF-NILs strategy confined the QTL to about 380 kb region supported by repeated genotype and phenotype data, and it lays the foundation for QTL cloning in the future. In addition, introgression of the QTL to an elite *japonica* variety SD785 was performed by successive backcrossing, and it confirmed the value of *qWS5* in increasing stem diameter and other agronomic traits during rice breeding.

**Conclusions:**

We prove that *qWS5* is a novel QTL with relatively stable effect for stem diameter and the QTL can be finely mapped to small region by the HIF-NILs strategy. The result will facilitate the improvement of rice lodging resistance by molecular marker assisted selection breeding.

**Supplementary Information:**

The online version contains supplementary material available at 10.1186/s12284-024-00723-x.

## Background

Lodging is a key problem in rice production, which caused loss of both yield and grain quality and difficulty in mechanical harvest (Islam et al. 2007; Setter et al. 1997). To improve the lodging resistance, the traditional method is to reduce the plant height. The widespread application of *sd1* gene has decreased lodging under nourishing fertilization by reducing plant height and led to improvements in the harvest index (Khush 1999). However, the semi-dwarf stature will reduce the total biomass of rice plants, and the rice yield improvement had reached a ceiling due to the biomass limitation (Okuno et al. 2014; Peng et al. 1999). To resolve the problem, different studies have tried to find new targets for lodging resistance without reducing the plant height. It was found that varieties with tall plant height still retained the ability of lodging resistance, and correlation analysis among traits revealed that stem diameter has stronger association with lodging resistance than plant height. The great contribution of stem diameter to lodging resistance was consistent both in rice and wheat and had been confirmed by analyzing more than 500 rice accessions (Guo et al. 2021; Kashiwagi et al. 2008; Zuber et al. 1999). Lower stem diameter value of basal internode was identified in lodging sensitive rice varieties (Hoshikawa and Wang 1990). Therefore, improvement of stem diameter has been recognized as an important target to increase rice lodging resistance both for direct seeding and transplanted systems (Guha et al. 2024). Moreover, strong culm is the important criteria for new plant type (NPT) rice, a concept raised by the IRRI breeder to break the yield bottleneck of semi-dwarf cultivars (Peng et al. 1999). It was confirmed that wider culm diameter with a thinner culm wall requires less investment of biomass and can maximize lodging resistance without reducing yield potential (Ookawa et al. 2010).

Like plant height, stem diameter is inherited in a quantitative manner but mainly controlled by minor QTLs. Stem diameter is susceptible to environmental change, and both nitrogen fertilizer level and planting density will affect the trait performance (Shah et al. 2019). Therefore, it is hard to dissect the underlying loci for stem diameter. Effective QTL dissection relies on suitable mapping populations. The primary populations including F_2_ and recombinant inbred lines (RILs) are usually used to map the coarse location of the QTL, while advanced populations including chromosome segment substitution lines (CSSLs) and nearly isogenic lines (NILs) are better for QTL validation and fine mapping (Bai et al. 2012; Yamamoto et al. 2009). High quality NILs have strong ability in isolating minor QTLs, and the QTL will express the characteristics of single mendelian factor in NILs even if it has small effect. In this way, three minor QTLs for panicle spikelet number have been clarified, and they explain more phenotypic variation compared with the performance found in RILs (Zhang et al. 2009). Heterogeneous inbred families (HIFs) are another way to develop the NILs, and NILs can be fast generated from available RILs (Tuinstra et al. 1997). Loci for heading date, panicle size and grain size have been dissected and even cloned by using HIF-NILs (Bai et al. 2017; Shao et al. 2010; Yan et al. 2011), indicating their critical value for genetic study. In the past decades, genetic study for stem diameter was popular and numerous QTL analyses were performed for the trait (Kashiwagi and Ishimaru 2004; Long et al. 2020; Chigira et al. 2023). The stem diameter QTLs were detected on different chromosomes, but most of them were identified only once. Similar results were revealed by several genome-wide association studies (GWAS) using natural populations (Guo et al. 2021; Meng et al. 2021; Sowadan et al. 2018). Though many QTLs for stem diameter were found either by linkage analysis or GWAS, most of them were not validated further, let alone subjected to gene cloning.

Several QTLs related to culm strength have been finely mapped and cloned, providing chance to further evaluate their breeding value and understand the underlying molecular mechanism. *Ghd7* is a pleiotropic QTL that mainly delays heading and increases plant height and panicle size, and it encodes a CCT domain protein. Interestingly, it also has large effect on stem growth, and NILs with *Ghd7* have wider stem (Xue et al. 2008). *SCM2* for strong culm was identified by the CSSLs, and is the first rice QTL cloned directly for the culm trait (Ookawa et al. 2010). *SCM2* is identical to the *APO1* gene encoding the F-box protein, which was previously cloned from mutant with aberrant panicle (Ikeda et al. 2007). Two QTLs for panicle structure share the similar *APO1* allele of *SCM2*, namely *qPL6* for panicle length and *PBN6* for panicle primary branch number (Terao et al. 2010; Zhang et al. 2015). Therefore, the superior *SCM2* allele has the potential to increase both grain yield and lodging resistance. *SCM3* is another strong culm QTL that is identical to the known *OsTB1* gene involving in the strigolactone signaling, and it also determines the panicle number and size (Yano et al. 2015). *qWS8* was cloned as a QTL from rice germplasm YYP1 with extremely strong culm, and is the major QTL widely used in China super hybrid rice to shape the NPT traits (Zhang et al. 2017). *qWS8* belongs to a novel allele of *IPA1* gene encoding the SBP-domain transcriptional factor, and was renamed as *ipa1-2D* in comparison to the previous *ipa1-1D* allele with strong effect for tiller number (Jiao et al. 2010; Zhang et al. 2017). *qPGN1* is a recently cloned QTL for peduncle stem diameter, and is identical to the *Gn1a* gene encoding the cytokinin oxidase, which is the well-known QTL for grain number control (Ashikari et al. 2005; Tu et al. 2022). Therefore, the null *gn1a* allele will not only increase the grain yield but also enhance the lodging resistance, and it also resides in the interval of *SCM1* locus for strong culm (Ookawa et al. 2010). *qSCM4* has been finely mapped to 58.5 kb region on chromosome 4, and it covers the cloned *Nal1* gene responsible for both leaf width and panicle size, which is also the underlying gene of QTL *SPIKE* (Fujita et al. 2013; Yang et al. 2023). *Nal1* is involved in the polar auxin transport and influences cell expansion and division (Qi et al. 2008). Another QTL called *GN4-1* was finely mapped to a different region of chromosome 4, and this QTL increases both grain number and diameter of upper culms, similar to the performance of *gn1a* (Zhou et al. 2018). However, the underlying gene for *GN4-1* is not verified. The above-mentioned researches reveal that QTLs involving strong culm may also have impact for the panicle development, suggesting that the organogenesis of stem and panicle is connected. The QTL cloning greatly facilitates our understanding about the molecular control of stem growth, and it lays the foundation to construct the regulatory pathway when more genes are characterized. Therefore, more QTLs for stem development need to be finely dissected to enhance both the theoretical and applied research of the trait.

In this study, we constructed different populations to thoroughly clarify the major QTLs for the wide stem performance of YYP1 variety, and focused on the fine-mapping of the novel QTL called *qWS5*. The effect of three major QTLs was validated both by F_2_ populations and RILs at two environments, and their combined contribution to stem and panicle traits was compared simultaneously. Fine mapping of *qWS5* was performed by using the recombinant progenies from HIF populations, and the QTL region was validated by different generations of NILs. We presented an effective strategy in delimiting important rice QTL sensitive to environments, and the *qWS5* region was narrowed down to about 380 kb in this way. We also explored the breeding value of *qWS5* by introgressing it into an elite *japonica* variety. The present research should lay the foundation for cloning the underlying gene of *qWS5* in the future.

## Result

### QTL Analysis of Stem Diameter Using F_2_ Populations at Different Sites

**Fig. 1:**
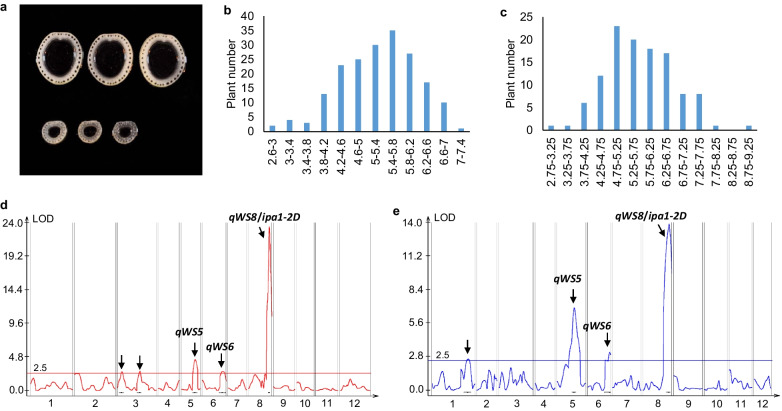
QTL analysis for stem diameter variation of strong culm variety YYP1. **a** Cross section of the internode III from parents YYP1 (upper) and NIP (lower). **b**, **c** The frequency distribution of stem diameter in F_2_ population planted in Hainan (**b**) and Shanghai (**c**). **d** QTL peak of stem diameter detected by F_2_ population in Hainan. **e** QTL peak of stem diameter detected by F_2_ population in Shanghai. Arrows indicate each QTL peak detected. Names of three QTL repeatedly detected at two sites were shown

The populations for QTL analysis were constructed by crossing NIP with YYP1. Compared with NIP, YYP1 has extremely strong culms, and therefore retains interesting loci for stem diameter control (Fig. 1a). We planted the F_2_ population from the two parents both in Hainan and Shanghai, two sites corresponding to tropical and temperate climates respectively. The trait at two sites showed the pattern of normal distribution and the overall stem diameter in Shanghai was larger than that in Hainan, suggesting that the trait is inherited in the quantitative manner and affected by environmental factors (Fig. 1b, c). We then performed QTL analysis to identify the loci for stem diameter at two sites. In Hainan, five QTLs were detected on chromosome 3, 5, 6, 8, and two QTLs were located on chromosome 3 (Fig. 1d and Table 1). In Shanghai, four QTLs were detected on chromosome 1, 5, 6, 8 (Fig. 1e and Table 1). Therefore, three QTLs were stably detected at both sites, namely *qWS5*, *qWS6* and *qWS8*. Among the three QTLs, *qWS8* has been cloned as a novel allele of *ipa1*, and renamed as *ipa1-2D* in our previous study (Zhang et al. 2017). Here we confirm that the QTL has greatest contribution to the stem diameter at different planting sites. *qWS6* is located at the same region of *qPL6*, and the two QTLs should be controlled by the same allele of *APO1*, which has been identified in our early research (Zhang et al. 2015).The LOD ratio of *qWS5* is smaller than *qWS8*/*ipa1-2D* but larger than *qWS6*, and *qWS6* can only be detected at the threshold level. Additive effect of *qWS5* and *qWS6*/*qPL6* is similar but significantly lower than that of *qWS8*/*ipa1-2D*, however, the dominant effect of *qWS5* is comparable to that of *qWS8*/*ipa1-2D* (Table 1). There is no report of QTL cloning for stem diameter trait in the *qWS5* interval, and it needs more investigation to learn about the new locus.

**Table 1:** The QTL information detected by F_2_ populations from two planting sites

QTL name	Detected sites	Chr	Interval of flanking markers	LOD values	Additive effect	Dominant effect	R^2^
*qWS3.1*	Hainan	3	782,964–7,233,841	2.7	0.02	0.44	0.03
*qWS3.2*	Hainan	3	24,866,320–28683508	2.8	0.19	− 0.14	0.05
*qWS5*	Hainan	5	22,247,059–26862749	4.4	0.28	0.18	0.03
*qWS6*	Hainan	6	25,832,443–29,886,117	2.7	0.26	0.02	0.04
*qWS8*	Hainan	8	24,779,537–25,649,013	23.4	0.78	0.26	0.24
*qWS1*	Shanghai	1	36,354,434–40,344,074	2.6	− 0.4	0.1	0.08
*qWS5*	Shanghai	5	18,016,584–22247059	6.9	0.3	0.57	0.03
*qWS6*	Shanghai	6	23,344,194–30,479,808	3.2	0.4	0.03	0.07
*qWS8*	Shanghai	8	25,034,644–26501151	13.9	0.81	0.35	0.2

R^2^ denotes the trait variations explained by the QTLs

### Single and Combined Effect of Three Major QTLs in RILs

The RILs and two parents were planted both in Yangzhou and Hainan, and Yangzhou has similar temperate climate like Shanghai. The trait values were obtained from all investigated plants for not only stem diameter but also panicle-related traits and plant height to clarify their correlation. The statistical analysis revealed that YYP1 has not only extraordinary stem diameter but also numerous panicle branches in comparison to NIP at both sites, and the trait values were greatly reduced in Hainan (Table 2). The performance of panicle length and plant height depended on the planting sites. The panicle length of YYP1 was comparable to that of NIP but its plant height was shorter than NIP in Yangzhou, however, both trait values from YYP1 were far more than that of NIP in Hainan. The RILs had large variations for all the five traits, and also showed great trait reduction when planted in Hainan (Table 2). Through Pearson correlation analysis, we found that stem diameter was significantly correlated with the remaining four traits, and the correlation became stronger in Hainan for all comparisons especially between stem diameter and plant height (Additional file 2: Table S2 and S3). The strongest correlations were identified between stem diameter and panicle branch number, which might be contributed by the pleiotropic genetic loci. All the remaining traits were significantly correlated with each other except for the comparisons of panicle primary branch number with panicle length or plant height in Yangzhou.

**Table 2:** The mean values of the parents and their derived RILs at two planting sites

Parents/RILs	SD (mm)	PL (cm)	PBN	SBN	PH (cm)
YYP1_YZ	9.01 ± 0.49	22.35 ± 1.99	27 ± 1.41	80 ± 10	89.17 ± 4.02
NIP_YZ	4.89 ± 0.33	22.53 ± 1.32	11 ± 1.67	20 ± 4.29	97.67 ± 4.27
*p*-value	9.1 × 10^–9^	0.855	6.4 × 10^–9^	9.5 × 10^–8^	5.3 × 10^–3^
YYP1_HN	7.77 ± 0.75	20.73 ± 1.76	22.33 ± 1.37	60.5 ± 13.79	81.17 ± 3.83
NIP_HN	2.8 ± 0.36	15.22 ± 0.98	4.83 ± 0.75	8.17 ± 2.04	67.17 ± 5.66
*p* value	4.4 × 10^–8^	5.4 × 10^–5^	9.4 × 10^–11^	3.4 × 10^–6^	5.3 × 10^–3^
RILs_YZ	6.42 ± 1.3	23.07 ± 2.89	16.63 ± 4.97	40.59 ± 20.36	98.67 ± 18.02
RILs_HN	5.19 ± 1.08	20.62 ± 2.69	10.79 ± 3.51	30.73 ± 14.89	84.46 ± 16.83
*p*-value	6.2 × 10^–14^	7.9 × 10^–11^	2.2 × 10^–21^	2.8 × 10^–5^	1.5 × 10^–9^

SD, PL, PBN, SBN and PH are abbreviations of stem diameter, panicle length, panicle primary branch number, panicle secondary branch number and plant height respectively. YZ and HN are abbreviations of two planting sites (Yangzhou and Hainan)

**Fig. 2:**
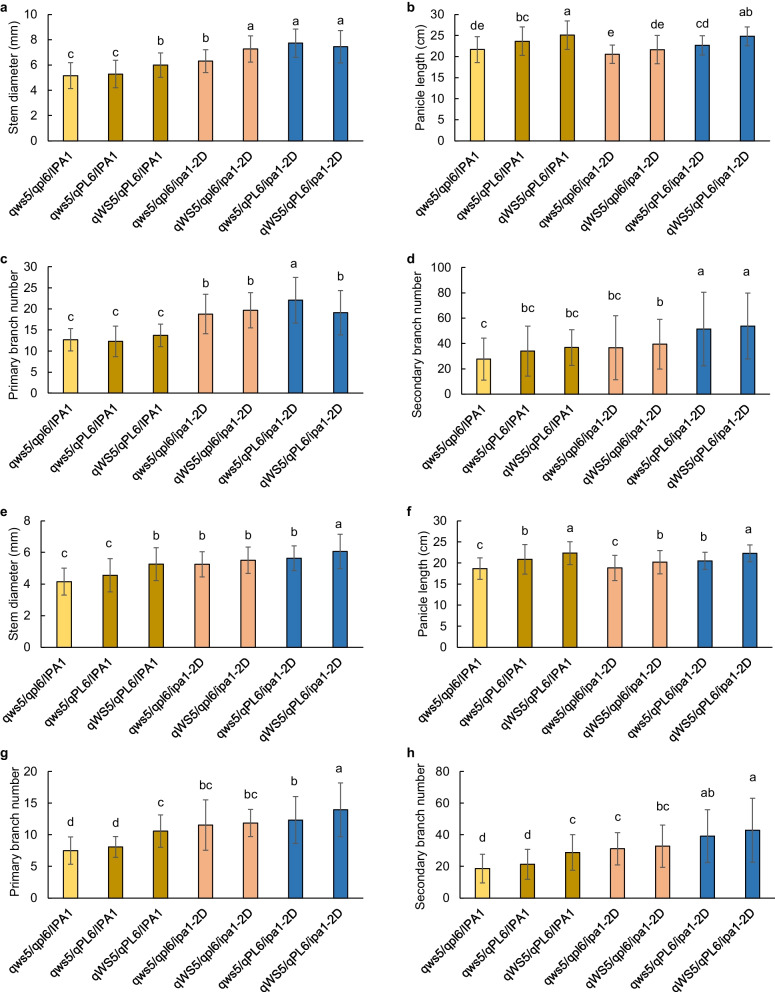
Trait performance of seven genotype combinations of *ipa1-2D*, *qPL6* and *qWS5* from RILs. **a**–**d** Comparison of stem diameter, panicle length, panicle primary branch number and secondary branch number in Yangzhou. **e**–**h**, Comparison of stem diameter, panicle length, panicle primary branch number and secondary branch number in Hainan. Values are means ± SD. Different letters indicate the significant difference between columns calculated by Tukey test

To clarify the contribution of three major QTLs in shaping the trait variation, the combinations of *qWS5*, *qPL6* and *ipa1-2D* homozygous genotypes among the RILs were clarified by the linked markers (Additional file 2: Table S1). All genotype combinations were identified from the RILs, namely, *qws5*/*qpl6*/*IPA1*, *qWS5*/*qpl6*/*IPA1*, *qws5*/*qPL6*/*IPA1*, *qWS5*/*qPL6*/*IPA1*, *qws5*/*qpl6*/*ipa1-2D*, *qWS5*/*qpl6*/*ipa1-2D*, *qws5*/*qPL6*/*ipa1-2D* and *qWS5*/*qPL6*/*ipa1-2D*. At least 11 lines were found for each genotype except for *qWS5*/*qpl6*/*IPA1*, which had only two lines and was excluded for trait comparison. Nevertheless, the rest genotype combinations still facilitate the comparison of either single or combined loci reciprocally, and we compared the difference of stem and panicle traits among the seven genotypes (Fig. 2). At both sites, the *ipa1-2D* stably increased the stem diameter compared with *IPA1* when they share the same genotypes of other loci, confirming the great contribution of this locus. However, *qPL6* only showed significant increase when combined with *qws5*/*ipa1-2D* in Yangzhou and *qWS5*/*ipa1-2D* in Hainan. *qWS5* increased the stem diameter significantly except for the combination with *qPL6*/*ipa1-2D* in Yangzhou and *qpl6*/*ipa1-2D* in Hainan (Fig. 2a, e). Therefore, the contribution of stem diameter either by *qPL6* or *qWS5* will be affected easily by the environments or genetic backgrounds. Nevertheless, pyramiding of *qPL6* and *qWS5* showed comparable contribution to stem diameter like that of *ipa1-2D* both in Yangzhou and Hainan. Pyramiding of either *qPL6* or *qWS5* with *ipa1-2D* can further improve the stem diameter only in Yangzhou, while pyramiding of three loci can greatly improve the stem diameter than any other genotypes in Hainan (Fig. 2a, e).

For panicle length, we found little contribution of *ipa1-2D* to the trait. *qPL6* steadily increased panicle length for any comparisons at both sites, confirming that *qPL6* is the major QTL for the trait. *qWS5* significantly increased the panicle length for most conditions except for its combination with *qpl6*/*ipa1-2D* at Yangzhou. When *qPL6* and *qWS5* were combined together, the panicle length was further increased (Fig. 2b, f). For panicle primary branch number, *ipa1-2D* can steadily increase the trait especially in Yangzhou, and contribution of *qWS5* and *qPL6* to this trait can be hardly found in Yangzhou. In Hainan, pyramiding of *qWS5* and *qPL6* showed similar performance like *ipa1-2D*, and pyramiding of all three loci showed the most panicle primary branch number (Fig. 2c, g). For panicle secondary branch number, it seems that contribution of either *ipa1-2D* or *qPL6* relies on each other, and the trait increase is significant when both loci exist. Little contribution to this trait can be found for *qWS5* (Fig. 2d, h). Therefore, all three loci have impact to the panicle traits, and the contributions of *ipa1-2D* to panicle primary branch number and *qPL6* to the panicle length are stable to environmental change. We did not find stable contribution of three QTLs in determining plant height, and significant difference was hardly found among the genotype combinations in Yangzhou (Additional file 1: Fig. S1). However, it seemed that the lines with two or three QTLs had the potential to increase the plant height in Hainan, which might explain the enhanced correlation between stem diameter and plant height (Additional file 1: Fig. S1 and Additional file 2: Table S3).

### Coarse Mapping of *qWS5* by RILs and HIF Populations

**Fig. 3:**
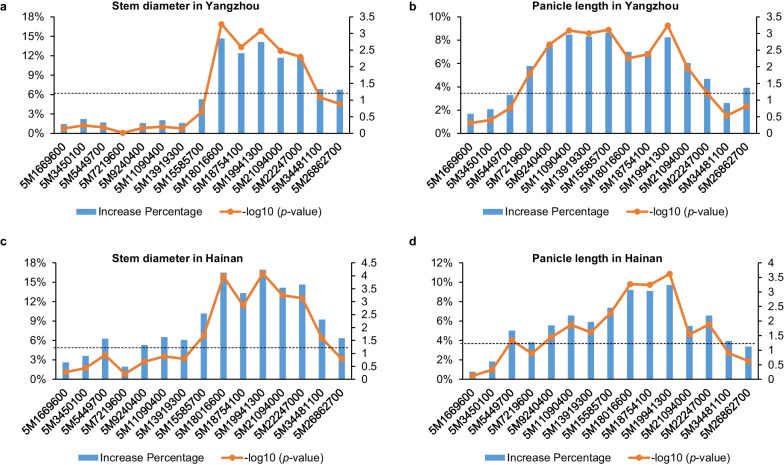
Single marker analysis of QTL effect by the RIL population. **a**, **b** The effect of different markers in determining the trait change of stem diameter (**a**) and panicle length (**b**) in Yangzhou. **c**, **d** The effect of different markers in determining the trait change of stem diameter (**c**) and panicle length (**d**) in Hainan. The left and right Y axis indicates the increase percentage and −log_10_ (*p* value) respectively. Dashed lines indicate the position of statistical level at *p* value of 0.05

To get a further understanding about the novel locus *qWS5*, we performed the coarse mapping taking advantage of the RIL population first. Fifteen polymorphic markers evenly distributed on chromosome 5 were selected to genotype the RILs (Fig. 3 and Additional file 2: Table S1), and most of the lines bear homozygous genotypes of the markers. Two groups of lines were generated based on the marker genotype, and we compared the difference of stem diameter and panicle length between the groups for each marker at two planting sites (Fig. 3a–d). If significant trait difference was identified between YYP1 allele and NIP allele of some markers, these markers should cover the region for the trait control. For stem diameter trait in Yangzhou, we found a marker interval with more than 10% trait promoting rate, which is located between markers 5M15585700 and 5M34481100, and five markers within the region showed the significant statistical level with –log_10_ (*p*-value) larger than 2 (*p* < 0.01) (Fig. 3a). Similar marker contribution was found for the trait in Hainan, confirming the location of the QTL (Fig. 3c). For the panicle length, a larger span of genomic regions with more than 6% promoting rate was identified, and two significant statistical peaks can be found, which cover the interval of 5M7219600-5M18016600 and 5M18754100-5M21094000, respectively. It suggests two QTLs for panicle length might reside in the long region. However, we found the peak on the left region became weak in Hainan condition, suggesting that the locus was sensitive to the environment change. Based on the changing rate of two traits, it can be inferred that the linkage region has larger contribution to stem diameter than panicle length.

**Fig. 4:**
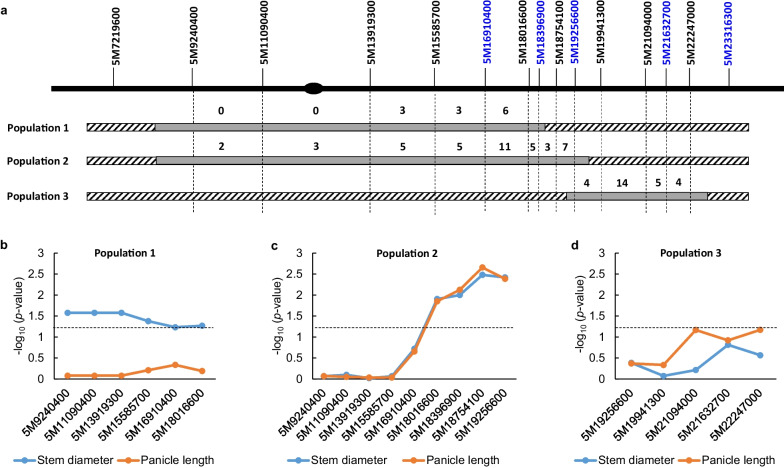
Confirmation of *qWS5* location by three F_8_ HIF populations. **a** The segregating segments of three populations. The markers used for genotyping and recombination number between markers for each population were indicated. Blue color denotes the five newly-developed markers. The ellipse denotes the position of centromere. The gray boxes and mottled boxes indicate segregated and fixed regions respectively. **b**–**d** The contribution of different markers to the trait variations of stem diameter and panicle length in population 1 (b), population 2 (**c**) and population 3 (**d**). The statistical significance was transferred as −log_10_ (*p* value) to facilitate illustration. The dashed lines denote the statistical significance at *p* value of 0.05

The interval mapped by primary populations needs to be clarified by the advanced populations without background noise. We then selected three F_8_ HIFs segregating at different regions of chromosome 5 but fixed at *qPL6* and *ipa1-2D*, which were planted as three populations, namely population 1 (P1), population 2 (P2) and population 3 (P3). Five new InDel markers were developed to delineate the border of the segregating chromosomal segments and find new recombinants (Fig. 4a). Six, nine and five markers were used to genotype each individual of three populations respectively, and statistical significance of each marker contributed to the trait variations was obtained (Fig. 4b–d). In P1, we found only 12 recombinants among the six markers, and no recombinant was found for three markers locating around the centromere. All the six markers have significant effect for stem diameter but little effect for panicle length (Fig. 4b). In P2, more recombinants were identified among the nine markers, and four successive markers on the right part showed large contribution to variations of both stem diameter and panicle length (Fig. 4c). For P3, no significant effect of the five markers was found for the two traits (Fig. 4d), suggesting the QTL was not harbored by the marker interval. The result indicates that the QTL for stem diameter is located in the marker interval shared by P1 and P2. Taking consideration of the interval identified by the RIL population, the locus for stem diameter was confined to about 3168 kb between markers 5M15585700 and 5M18754100. Moreover, the recombinant progenies of populations P1 and P2 laid the foundation to further narrow down the QTL.

### Fine Mapping of *qWS5* to ~ 380 kb by HIF-NIL Strategy

**Fig. 5:**
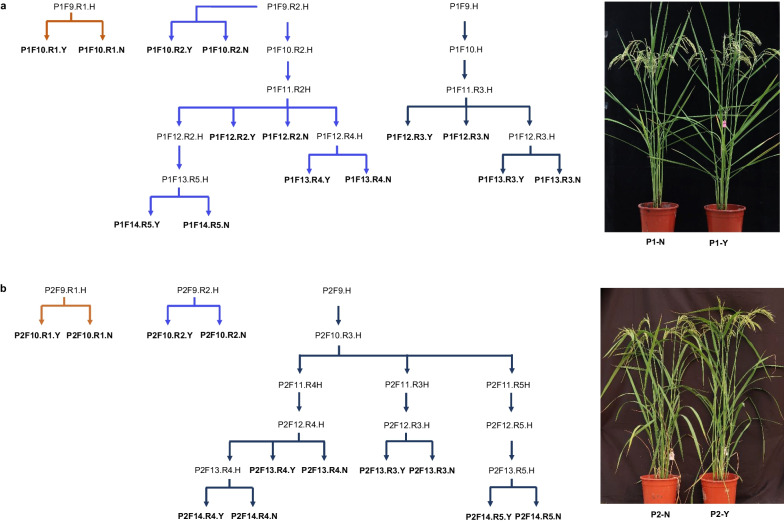
The sketch map illustrating the pedigree of HIF-NILs from population 1 (**a**) and population 2 (**b**). Arrows with the same color designate the same origination from respective heterozygous F_8_ plants. The bold characters indicate the HIF-NILs for trait comparison. It can be seen on the right panel that the either P1 or P2-derived NILs are highly similar for the whole morphology

To accurately dissect the *qWS5* region, we developed a set of HIF-NILs with different recombination sites from F_9_ to F_14_ generations of P1 and P2 population. During the process, if a heterozygous plant was identified with new recombination, it was selfed to generate homozygous sister lines, namely, HIF-NILs. Single-seed descent method was used to generate heterozygous plants at different generations, and new recombination lines with purified background can be found and selfed to obtain the new HIF-NILs. In this way, seven and six pairs of HIF-NILs were developed from P1 and P2 populations respectively, and we found the overall morphology of HIF-NILs was highly similar (Fig. 5).

**Fig. 6:**
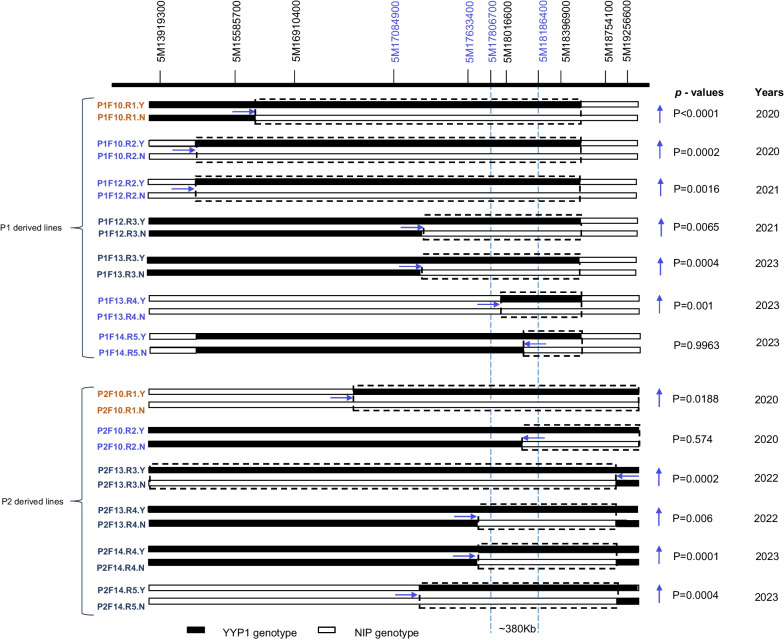
Fine mapping of *qWS5* to about 380 kb by the HIF-NIL strategy. The genotypes of HIF-NILs with different recombination sites were shown by black (YYP1) and white (NIP) bars. Marker names with blue color indicated the newly-developed one. The upper and lower panels indicated the P1 and P2-derived lines respectively. Dashed boxes indicated the intervals with contrasting genotypes. The *p* values for comparison of each pair of NILs were shown on the right. Vertical arrows indicate the changing trends of stem diameter by comparing YYP1 genotype with NIP genotype. Horizontal arrows denote the directions of target QTL location based on the trait change between NILs. Different years for trait evaluation were shown on the right

The HIF-NILs were planted in Yangzhou at different years for trait comparison (Fig. 6). For P1 pedigree, P1F10.R1 harbors the recombination site between markers 5M15585700 and 5M16910400, P1F10.R2/P1F12.R2 shared the recombination site of the same origin between markers 5M13919300 and 5M15585700, and P1F12.R3/P1F13.R3 shared the recombination site of the same origin between markers 5M17084900 and 5M17633400. The trait comparison showed that above-mentioned NILs with YYP1 segments will increase the stem diameter at statistical significance of *p* < 0.01, indicating regions of the contrasting genotype contain the QTL. Accordingly, *qWS5* was definitely located at the right of marker 5M17084900. P1F13.R4 and P1F13.R5 had new recombination sites between markers 5M17806700 and 5M18016600 and between markers 5M18016600 and 5M18186400, respectively. The R4 NIL with YYP1 segment showed increased stem diameter, locating *qWS5* further to the right of 5M17806700. No difference can be found between R5 NILs, and it means that the *qWS5* resides in the non-segregating region on the left of marker 5M18186400. Therefore, *qWS5* was finally mapped to the interval between marker 5M17806700 and 5M18186400, which is about 380 kb (Fig. 6). Similar comparison was performed for lines from P2 pedigree. P2F10.R1 and P2F10.R2 planted in year 2020 confined the QTL between marker 5M16910400 and 5M18186400, and P2F13.R3 and P2F13.R4 planted in year 2022 further delimited the QTL to the right of marker 5M17633400, which was further validated in year 2023 by P2F14.R4 and P2F14.R5 (Fig. 6). We also compared the trait of panicle length for the HIF-NILs, and found the trait variation is not as significant as that of stem diameter, and some HIF-NILs did not showed statistical significance (Table S4), suggesting the region has weak effect for the trait. Nonetheless, we successful mapped the stem diameter QTL to a small region and provided a quotable strategy to accurately map QTLs.

### Evaluating the Breeding Value of *qWS5 *in Elite *Japonica* Variety Background

**Fig. 7:**
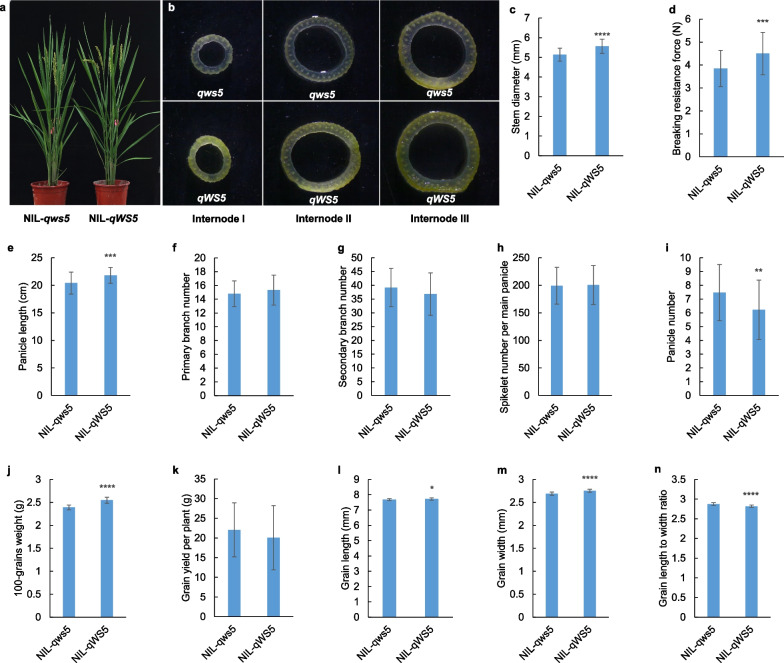
Testing the breeding value of *qWS5* in the background of elite *japonica* variety SD785. **a** Overall plant morphology of the NILs. Note that the lines showed no obvious difference for plant height and heading date. **b** Cross-sections of culms from the first internode to the third internode of the NILs. **c**–**n**, comparison of twelve traits between NILs, including stem diameter, breaking resistance force, panicle length, panicle primary branch number, panicle secondary branch number, spikelet number per main panicle, panicle number, 100-grains weight, grain yield per plant, grain length, grain width and grain length to width ratio. Values are means ± SD; n = 48 plants. The statistical significances with *p* < 0.05, *p* < 0.01, *p* < 0.001 and *p* < 0.0001 were highlighted by *, **, *** and **** respectively

We also tested the value of *qWS5* in breeding practice. By successive backcrossing, we obtained a pair of high-quality NILs covering marker interval of 5M16910400 and 5M18396900 at the BC_7_F_3_ generations in the background of SD785, the elite *japonica* rice variety with high yield potential but weak lodging resistance. We found the NILs shared similar overall plant morphology, and the heading date and plant height were identical (Fig. 7a). When the culms were compared, we found the *qWS5* line had larger stem diameter at all the internodes (Fig. 7b). At the mature stage, we measured both the stem diameter and some other important agronomic traits to get a full view about the QTL in SD785 background (Fig. 7c–n). The result showed that introgression of *qWS5* region greatly increased the stem diameter, which in turn enhanced the breaking resistance of the culms (Fig. 7c, d), indicating an enhanced ability for lodging resistance. The panicle length was also increased, but no difference can be found for panicle primary branch number, panicle secondary branch number and spikelet number per main panicle (Fig. 7e–h). NIL-qWS5 has less panicle number but higher grain weight. The grain weight increase might balance the negative effect of reduced panicle number for yield potential, as no significant difference was found for the grain yield per plant (Fig. 7i–k). To learn what grain dimensions determine the grain weight, we also characterized the grain shape change between the NILs, and found that both the grain length and grain width were increased in the NIL-qWS5, with larger effect for grain width. Therefore, the grain length to width ratio was reduced (Fig. 7l–n). The trait analysis indicated that *qWS5* was an ideal breeding target to be utilized for lodging resistance, and it has little negative effect for yield potential.

## Discussion

The stem diameter has been recognized as the important criteria for lodging resistance, and many genetic studies have been conducted to explore the underlying loci of the trait. Ninety-eight backcrossed inbred lines from NIP and *aus* variety Kasalath have been used to dissect the stem diameter QTLs, and six loci were identified on chromosomes 1, 3, 6, 7, 8, 12 respectively. All the QTLs are contributed by the Kasalath parent except for the one locating on chromosome 3 (Kashiwagi and Ishimaru 2004). Another study clarified the QTLs for stem diameter across different planting sites and years by using the BC_2_F_20_ lines derived from the cross of *indica* variety 9311 and wild rice (*Oryza longistaminata*), and four QTLs locating on chromosomes 1, 8, 9 and 12 were detected, all of which were contributed by the wild rice (Long et al. 2020). Most recently, QTL analysis were performed by RILs from cross between two *japonica* varieties Omachi and Koshihikari, and three QTLs for culm diameter were identified on chromosomes 3 (*qCD3*) and 7 (*qCD7-1* and *qCD7-2*) (Chigira et al. 2023). However, most of the QTLs cannot be stably detected among years and locations, indicating that environment will greatly affect the QTL performance for stem diameter. In addition to linkage analysis, GWAS was also employed to explore the genetic loci for stem diameter. 521 rice cultivars have been genotyped by 262 SSR markers in two years, and 14 QTLs associated with stem diameter were identified on chromosomes 1, 4, 5, 6, 7, 8, 10 and 11 (Sowadan et al. 2018). Recently, a GWAS study with high-density SNP markers was performed to dissect the loci for lodging-related traits among 524 rice accessions, and the loci for stem diameter were identified on chromosomes 1, 3, 4, 5, 6, 7, 9, 10 and 11. Some of the loci were specially detected in the panel belonging to *indica* or *japonica* subspecies, and the locus on chromosome 5 was only detected once in the *japonica* panel for the outer stem diameter of second internode (Guo et al. 2021). Another GWAS study investigated 550 accessions both for outer and inner stem diameter, and found no QTL for outer diameter but 17 QTLs for inner diameter distributing on all chromosomes except for chromosome 4 and 11 (Meng et al. 2021). Therefore, the stem diameter trait is controlled by many different genetic loci and sensitive to the environmental change.

In this study, we performed QTL analysis using F_2_ populations derived from the cross of NIP and YYP1, a landrace with extremely strong culm, and three stable QTLs were detected on chromosome 5, 6 and 8 at different years and locations. *qWS8* had the greatest contribution to stem diameter and had been cloned as a novel allele of *ipa1*, and was renamed as *ipa1-2D* (Zhang et al. 2017). *qWS6* was co-located with *qPL6* for panicle length, which was allelic to the strong culm QTL *SCM2* (Ookawa et al. 2010; Zhang et al. 2015). Comparing the location of *qWS5* with previously identified loci, we found that *qWS5* is a novel QTL never identified before, and it may contain the rare allele specifically shared by landraces like YYP1, similar to the condition of *ipa1-2D* (Zhang et al. 2017). In parallel comparison of three QTLs in RILs confirmed that *ipa1-2D* has the largest and stable contribution to both stem diameter and panicle primary branch number at any conditions. Though *qPL6*/*SCM2* has been cloned as an important QTL for lodging resistance, we found the contribution of the QTL to stem diameter is not as significant as that to panicle length. Therefore, *qPL6*/*SCM2* could be a major QTL for panicle length but minor QTL for stem diameter. The effect of *qWS5* for stem diameter was larger and stabler than that of *qPL6*/*SCM2*, and it could be an ideal target to be selected for lodging resistance in rice breeding. Importantly, the combination of *qWS5* with other two loci will generate gradient performance of stem diameter, making it possible to design the trait compatible to different genetic background and environments.

Due to the genetic background noise, QTL fine mapping can be hardly realized by primary mapping population like F_2_ and RILs, and NILs are the best way to precisely estimate the QTL effect, which separate the QTL as a mendelian factor without background noise. The general way of NIL development is to perform several rounds of backcrossing and a round of selfing to generate homozygous sister lines with contrasting genotype at target QTL. However, it will take additional three years to finish the step after QTL information is obtained, which is time consuming (Bai et al. 2012). HIF analysis provides an alternative way to fast generate NILs from inbred populations of early or advanced generations, and the NILs will be more homogeneous if the populations are more inbred (Tuinstra et al. 1997). Theoretically, more than 98% of genetic background will be homozygous from F_7_ inbreds, and several QTLs for grain size have been successfully fine-mapped at such generation (Bai et al. 2017; Shao et al. 2010). In our study, we proved that HIF-NILs also worked well for stem diameter trait. We updated the process for QTL fine mapping using HIF-NILs, and the new recombinants were identified directly from previous recombinant heterozygous lines in the single-seed descent way, which can further exclude the disruption of potential background noise. During the process, we chose new NILs to test its effect in new season, which will reduce the risk of misjudgment due to genetic background and environmental fluctuation. We believe that this strategy will generate high-quality NILs and therefore facilitate the dissection of QTL sensitive to environments.

NILs are also ideal materials to accurately evaluate the contribution of QTLs, and the allele effect of several stem diameter QTLs has been clarified accordingly. NIL114 and NIL28 are two NIP NILs with either single QTLs on chromosome 8 or four QTL on chromosomes 1, 7, 8, 12 delivered by Kasalath. It showed that the two lines could increase the stem diameter by about 7% and 39% respectively, but NIL28 had taller plant that is negative for lodging resistance (Kashiwagi et al. 2008). High-quality NILs have been developed for *SCM2* and *SCM3*, and the comparison of two NILs showed that *SCM3* had bigger effect for stem diameter than *SCM2*, and when the two loci were pyramided, the stem diameter were further enlarged (Yano et al. 2015). Recently, the NILs of *qPND1* and *SCM2* were developed respectively, and the trait comparison showed that two loci function additively to increase stem diameter, with a 14.08% increase for *qPND1*, and 8.09% increase for *SCM2*, and a 23.31% increase for two loci together (Tu et al. 2022). Later, NILs for *SCM1* to *SCM4* and their pyramiding lines were developed in the Koshihikari background and the stem trait increased incrementally when more QTLs accumulated. Nearly all the NILs could avoid lodging except for NIL-*SCM2*, and NILs with four QTLs showed greatest increase of basal stem diameter compared with the control line (Ookawa et al. 2022). *qSCM4* was identified as a QTL for strong culm on chromosome 4 and NIL carrying *qSCM4* had a 25.3% increase for basal stem diameter (Yang et al. 2023). In our study, we can make the judgement directly from different pairs of HIF-NILs, and found that the YYP1 allele of *qWS5* will increase the stem diameter by about 8%-14% among different HIF-NILs, and this effect is similar to that revealed by the RIL population and NILs of SD785 background. Our previous research showed that the weak allele of *Sd1* will increase the stem diameter by about 16% compared with the null allele in the Daohuaxiang background, and such allele variation also exists in the YYP1/NIP population (Zhang et al. 2020a). However, we only identified the QTL for stem diameter at the *Sd1* location in Shanghai but not Hainan, and it suggests the effect of *Sd1* on stem diameter might be affected by the genetic background and environment. Therefore, *qWS5* is a QTL with relatively stable effect for stem diameter and could has larger contribution to lodging resistance application during rice breeding.

Though some QTLs have been identified to contribute greatly to the stem diameter, most of them showed pleiotropic effect on other traits, which might counteract the effect for lodging resistance and yield improvement. Correlation analysis of different traits in rice germplasms showed that the culm strength traits were positively correlated with panicle weight but negatively correlated with panicle number (Guo et al. 2021). This is consistent with the performance of many cloned QTLs for stem diameter. The *ipa1-2D* locus increased both the stem diameter and panicle size, but reduced the panicle number (Zhang et al. 2017). Differently, *qPL6*/*SCM2* locus increased the stem diameter and panicle size, and had no negative effect for panicle number (Zhang et al. 2015). Compared with *SCM2*, *SCM3* had weaker effect for panicle size but greatly reduced the panicle number. Interestingly, the reduction in panicle number could be mitigated by the pyramiding of *SCM2* and *SCM3*, suggesting a possible additive interaction of two loci (Yano et al. 2015). *qSCM4* had pleiotropic effect for stem diameter and panicle size, and it increased the panicle primary and secondary branch number by 14.7% and 9.9% respectively (Yang et al. 2023). However, *qSCM4* effect on panicle number was not explored. The QTLs described above have little effect on plant height, suggesting that they mainly affect the development of culm width. Nevertheless, *Sd1* involving the GA synthesis and other GA-related genes exerts functions for stem development by enhancing both culm diameter and length (Okuno et al. 2014; Ookawa et al. 2016). In our study, we tested the pleiotropic effect of *qWS5* on other agronomic traits related to yield in the SD785 background. The result showed that the locus had no effect on plant height but reduced the panicle number. However, the total yield was not affected as the locus increased the grain weight simultaneously. Mutation of grain size gene *GW2* leads to increased stem diameter (Yamaguchi et al. 2020), suggesting possible link of two traits during development. Our previous research also identified the QTL for grain length in the similar interval as *qWS5* (Zhang et al. 2020b), and QTL cloning should be performed in the future to clarify the pleiotropic effect. Nevertheless, the NILs developed in this study could be an ideal breeding donor for *japonica* rice breeding in Jiangsu Province of China, as the SD785 is an elite *japonica* variety released in recent years. The *qWS5* locus can be fast introgressed to other elite *japonica* varieties by the NILs, and therefore alleviates the severe lodging problem encountered by most varieties in recent years.

## Conclusions

Our result dissected the major QTLs for the strong culm morphology, and clarified the single and combined contribution of *ipa1-2D*, *qPL6* and *qWS5* to the trait at two planting sites. We proved that *qWS5* is a novel QTL with relatively stable effect for stem diameter and successfully narrowed down the QTL to a small region by the HIF-NILs strategy. The present result will facilitate the cloning of the *qWS5* and its application in molecular marker assisted selection breeding in future. Indeed, we found that introgression of *qWS5* significantly improved the lodging resistance of the elite *japonica* variety SD785, which didn’t affect the yield potential.

## Materials and Methods

### Populations Developed for QTL Mapping

F_1_ plants were generated by crossing NIP with YYP1, and F_2_ populations were obtained from the F_1_ plants. The F_2_ individuals were planted in Hainan and Shanghai to perform the QTL analysis respectively. Starting from the F_2_ generation, the seeds of each plant were harvested and planted for successive selfing in the single-seed descent way. 119 independent F_6_ lines were obtained and planted as RILs in Yangzhou to compare the single or combined effect of three major QTLs, and the F_7_ lines were planted again in Hainan to compare the environmental effect. Then, three F_8_ populations segregating at the potential *qWS5* regions were developed by selecting the F_7_ RILs, and planted in Yangzhou to perform linkage analysis. To narrow down the QTL region, several recombinant heterozygous plants were selected from F_8_ population 1 and population 2 for further selfing and sister lines with YYP1 and NIP alleles from the single heterozygous plants at F_10_, F_12_, F_13_ and F_14_ generations were obtained and planted as NILs in Yangzhou for trait comparison in different years. The pedigrees of different sister lines are illustrated in the result section. To develop the *qWS5* NILs in the background of elite variety SD785, the plant with *qWS5* region was crossed with SD785 and then backcrossed with the recipient for seven times, and BC_7_F_1_ plants were obtained and selfed to generate segregating BC_7_F_2_ plants. One pair of BC_7_F_3_ sister lines with YYP1 and NIP alleles at the QTL region were selected and planted in Yangzhou for trait evaluation.

### Trait Evaluation of Different Lines

Different populations and lines were sown in nursery beds and later transplanted to the field under irrigated condition with normal water and fertilizer management. Stem diameter and panicle traits including panicle length and panicle branch number were evaluated from the main culms, which were labeled at the seedling stage to facilitate accurate finding. At the mature stage, the main culms were cut from the base to measure the traits. The stem diameter was measured as the values of major axis and minor axis at the central part of the third internode by using vernier caliper, and the mean of two values was used as the final data. The panicle length was measured by the rulers from the panicle tip to the peduncle base, and the plant height was measured as the distance from the panicle tip to the plant base. The primary and secondary branch number of main panicle were counted manually. Six plants of each RIL were investigated for different traits. More agronomic traits were evaluated for the SD785 NILs. In addition to the traits described above, the panicle number and spikelet number per main panicle were counted manually. All seeds from a plant were weighted as the grain yield per plant. The 100 grains were counted and weighted as the 100-grains weight. The grain shape traits were investigated by the automatic seed analysis system (SC-G, Hangzhou wanshen, China). The main culm was used to test the breaking resistance force, and the breaking force against the central part of the third internode was recorded by the stem strength tester (YYD-1A, Zhejiang Tuopu, China). To compare the cross section of stems, the main culm was cut into slices by hand and observed by the stereoscope. In addition, Pearson correlation coefficient was calculated between any two traits investigated for RILs to learn the trait correlation.

### Linkage Analysis of Different Populations and NILs

190 and 119 F_2_ plants planted in Hainan and Shanghai were genotyped by 145 and 123 polymorphic markers respectively, and marker genotypes of each plant were recorded. The QTL analysis was performed by software WinQTLCART 2.5 after inputting the genotype and phenotype information. The threshold for QTL detection was set as 2.5 empirically. Markers linked with three major QTLs were used to genotype the F_6_ RILs and lines with homozygous genotype combinations were subjected to trait comparison. Tukey test was used to perform multiple comparisons of different combinations. To confirm the *qWS5* interval, markers evenly distributed on chromosome 5 were selected and used to genotype the RILs, and the means of two homozygous genotypes and the statistical significance for each marker were calculated. The increase percentage was calculated as follows: (YYP1 values − NIP values)/NIP values. −log_10_ transformations were performed for the *p*-values to facilitate the illustration of marker effect. Similarly, three F_8_ HIF populations segregating at different regions of chromosome 5 were selected and 96 plants from each population were genotyped by the markers distributed in the region. Contributions of different markers were obtained by comparing trait variations between homozygous genotypes. New markers were designed in the target region when necessary. Some markers were also used to genotype different generations of HIFs from F_9_ to F_14_ to obtain the sister lines as NILs, and the regions with contrasting alleles between the sister lines were validated accordingly. 14 plants from each sister line were subjected to trait comparison, and the region with contrasting alleles should cover the QTL if the traits have significant difference. The statistical significances of comparisons between homozygous genotypes described above were calculated by the student’s t-test.

### Supplementary Information

**Additional file 1: Fig. S1**. Variations of plant height among seven genotype combinations of *ipa1-2D*, *qPL6* and *qWS5* from RILs in Yangzhou (a) and Hainan (b).

**Additional file 2: Table S1**. Markers used in this study for linkage analysis of *qWS5*, *qPL6* and *ipa1-2D*.** Table S2**. Correlation of five traits from RILs in Yangzhou.** Table S3**. Correlation of five traits from RILs in Hainan.** Table S4**. The trait values of HIF-NILs with different segments at different generations.

## Data Availability

The datasets supporting the conclusions of this article are included within the article and additional files.

## Abbreviations

QTL: Quantitative trait loci
HIF: Heterogeneous inbred family
RIL: Recombinant inbred line
NIL: Nearly isogenic line
kb: Kilobase
GWAS: Genome-wide association studies
InDel: Insertion and deletion
NPT: New plant type
CSSL: Chromosome segment substitution line
NIP: Nipponbare
