# Supplementary material for: Genetic Dissection of Major Rice QTLs for Strong Culms and Fine Mapping of qWS5 for Breeding Application in Transplanted System
Source: Rice (N Y). 2024 Jul 12;17:43. doi: 10.1186/s12284-024-00723-x (PMC11245457; doi:10.1186/s12284-024-00723-x)
Supplement: Supplementary file 1 — Additional file 1: Fig. S1. Variations of plant height among seven genotype combinations of ipa1-2D, qPL6 and qWS5 from RILs in Yangzhou (a) and Hainan (b). [file 12284_2024_723_MOESM1_ESM.docx]

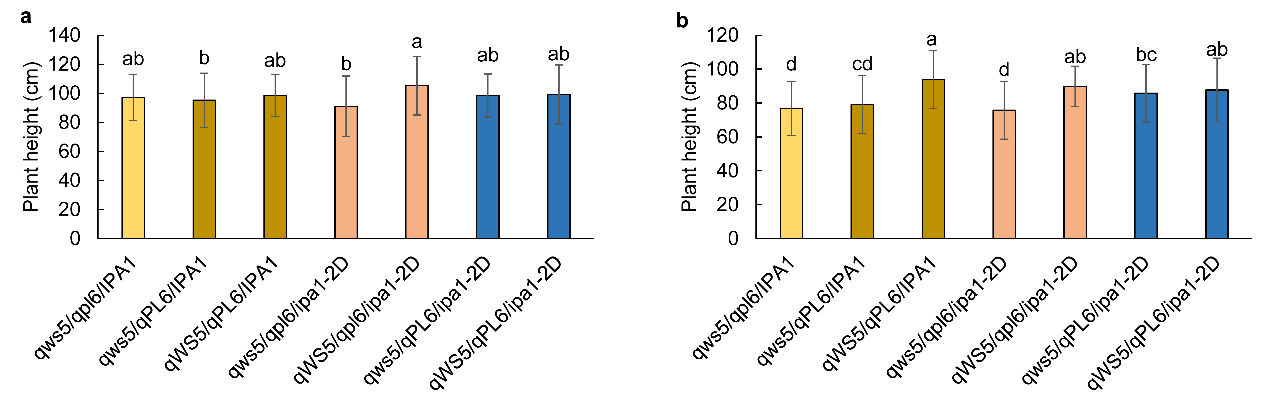


**Figure S1.** Variations of plant height among seven genotype combinations of *ipa1-2D*, *qPL6* and *qWS5* from RILs in Yangzhou (a) and Hainan (b). Values are means ± SD. Different letters indicate the significant difference between columns calculated by Tukey test.
